# Identifying Risk Factors for Prolonged Length of Stay in Hospital and Developing Prediction Models for Patients with Cardiac Arrest Receiving Targeted Temperature Management

**DOI:** 10.31083/j.rcm2402055

**Published:** 2023-02-06

**Authors:** Wei-Ting Chiu, Lung Chan, Jakir Hossain Bhuiyan Masud, Chien-Tai Hong, Yu-San Chien, Chih-Hsin Hsu, Cheng-Hsueh Wu, Chen-Hsu Wang, Shennie Tan, Chen-Chih Chung

**Affiliations:** ^1^Department of Neurology, Taipei Medical University - Shuang Ho Hospital, 235 New Taipei City, Taiwan; ^2^Department of Neurology, School of Medicine, College of Medicine, Taipei Medical University, 110 Taipei, Taiwan; ^3^Division of Critical Care Medicine, Department of Emergency and Critical Care Medicine, Taipei Medical University - Shuang Ho Hospital, 235 New Taipei City, Taiwan; ^4^Health Informatics Department, Public Health Informatics Foundation, 1216 Dhaka, Bangladesh; ^5^Department of Critical Care Medicine, MacKay Memorial Hospital, 104 Taipei Branch, Taiwan; ^6^Department of Internal Medicine, National Cheng Kung University Hospital, College of Medicine, National Cheng Kung University, 704 Tainan, Taiwan; ^7^Department of Critical Care Medicine, Taipei Veterans General Hospital, National Yang-Ming University, 112 Taipei, Taiwan; ^8^Coronary Care Unit, Cardiovascular Center, Cathay General Hospital, 106 Taipei, Taiwan

**Keywords:** cardiac arrest, hypothermia, IHCA, length of stay, OHCA, outcome, prediction, targeted temperature management

## Abstract

**Background:**

Prolonged length of stay (LOS) following targeted temperature management (TTM) administered after cardiac arrest may affect healthcare plans and expenditures. This study identified risk factors for prolonged LOS in patients with cardiac arrest receiving TTM and explored the association between LOS and neurological outcomes after TTM.

**Methods:**

The retrospective cohort consisted of 571 non-traumatic cardiac arrest patients aged 18 years or older, treated with cardiopulmonary resuscitation (CPR), had a Glasgow Coma Scale score <8, or were unable to comply with commands after the restoration of spontaneous circulation (ROSC), and received TTM less than 12 hours after ROSC. Prolonged LOS was defined as LOS beyond the 75th quartile of the entire cohort. We analyzed and compared relevant variables and neurological outcomes between the patients with and without prolonged LOS and established prediction models for estimating the risk of prolonged LOS.

**Results:**

The patients with in-hospital cardiac arrest had a longer LOS than those with out-of-hospital cardiac arrest (*p* = 0.0001). Duration of CPR (*p* = 0.02), underlying heart failure (*p* = 0.001), chronic obstructive pulmonary disease (*p* = 0.008), chronic kidney disease (*p* = 0.026), and post-TTM seizures (*p* = 0.003) were risk factors for prolonged LOS. LOS was associated with survival to hospital discharge, and patients with the lowest and highest Cerebral Performance Category scores at discharge had a shorter LOS. A logistic regression model based on parameters at discharge achieved an area under the curve of 0.840 to 0.896 for prolonged LOS prediction, indicating the favorable performance of this model in predicting LOS in patients receiving TTM.

**Conclusions:**

Our study identified clinically relevant risk factors for prolonged LOS following TTM and developed a prediction model that exhibited adequate predictive performance. The findings of this study broaden our understanding regarding factors associated with hospital stay and can be beneficial while making clinical decisions for patients with cardiac arrest who receive TTM.

## 1. Introduction

Sudden cardiac arrest is among the leading causes of premature death and disability worldwide [1, 2]. Cardiac arrest imposes a considerable clinical and economic burden on the healthcare system, society, families, and individual patients, including direct and indirect medical and nonmedical care costs, such as financial losses resulting from the loss of productivity. In the United States, the annual and lifetime economic productivity losses resulting from out-of-hospital cardiac arrest (OHCA) were estimated at US$11.3 and US$150.2 billion in 2018 [2]. The annual loss associated with cardiac arrest in Australia was estimated to be US$1.42 billion [3]. The estimated cost of medical care after OHCA is more than US$120,000 per patient in the United States [4, 5]. OHCA results in medical expenses of more than US$33 billion annually, and hospitalization costs account for 17% of the total medical cost [4, 5].

Many deaths associated with cardiac arrest occur before arrival at the hospital, and most patients hospitalized after the restoration of spontaneous circulation (ROSC) do not survive discharge [1, 6]. Those discharged with strong functional recovery are among the minority [1, 6, 7]. Despite the scientific debate, to reduce adverse outcomes associated with cardiac arrest, targeted temperature management (TTM) has been introduced to improve neurological recovery in patients with cardiac arrest [8, 9, 10, 11]. A longer length of stay (LOS) and the use of TTM or cardiovascular interventions after arrest significantly affect medical expenditure [4, 5]. LOS is a significant determinant of post-care costs, and identifying the independent predictors of LOS can help improve resource allocation and cost-effectiveness. Furthermore, longer LOS may reduce the quality and efficiency of healthcare. Prolonged LOS may affect the capacity of hospital beds and the availability of healthcare personnel to accommodate patients, which may decrease the healthcare quality and deny other patients access to inpatient care [12, 13, 14]. Extended hospital stays may also result in patients being more susceptible to hospital-acquired infections and lead to less favorable outcomes [12, 15]. In addition, the devices and procedures used to achieve TTM incorporate many complex protocols [16]. Significant heterogeneity exists in patients who received TTM [10, 16, 17]. The interventions and medications used in TTM vary greatly depending on the patient’s condition, and there may be different complications after TTM that require additional treatments [6, 10, 16, 18]. These variabilities may result in prolonged LOS for patients undergoing TTM. In addition to potentially increasing the time spent in the hospital, TTM may delay critical decision-making regarding whether to continue active treatment or withdraw care [19]. There have been many reports discussing and establishing the predictions of neurological outcomes of TTM [20, 21, 22, 23, 24, 25]. No study has determined the relationship between TTM outcomes and LOS in hospitals after cardiac arrest.

The present study identified risk factors for prolonged LOS in patients undergoing TTM, explored the association between neurological outcomes after TTM and LOS, and developed a predictive model to estimate the risk of prolonged LOS in patients with cardiac arrest before the application of TTM.

## 2. Materials and Methods

### 2.1 Patient Population

Clinical data from medical records obtained from the Taiwan Network of Targeted Temperature Management for Cardiac Arrest (TIMECARD) registry were retrospectively reviewed [20, 26]. The TIMECARD registry is a nationwide multi-center registry project conducted between January 2014 and September 2019 in nine medical centers in Taiwan [20, 22, 26]. Each participating hospital reported its patient-level data by using an online case report form. All electronic medical data were anonymized.

We included patients who were aged 18 years or more, experienced a nontraumatic cardiac arrest event that occurred inside or outside the hospital, were treated with cardiopulmonary resuscitation (CPR), had a Glasgow Coma Scale score of <8 or the inability to obey commands after ROSC, and underwent TTM less than 12 h after ROSC.

We excluded patients who experienced uncontrollable bleeding or intracranial hemorrhage; had impaired consciousness before cardiac arrest, as indicated by a Cerebral Performance Category (CPC) score of ≤3, regardless of etiology; had fatal ventricular arrhythmia; and had a life expectancy of fewer than 6 months.

### 2.2 Study Protocol

All eligible patients were treated in accordance with the TTM protocol following the consensus scientific statement issued by the Taiwan Society of Emergency and Critical Care Medicine [26]. TTM is designed to maintain a patient’s temperature within the target range with minimal variation. Depending on the clinical protocol of each participating hospital, TTM can be applied using cold saline infusion, cooling blankets, intravenous cooling catheters, extracorporeal membrane oxygenation, or in combination with other therapies [20, 26]. All protocols maintain the patient’s temperature at the target level for at least 24 h and rewarm them to normal body temperature at a relatively slow rate [20]. Patient registration data were recorded using the latest Utstein resuscitation registration template [26, 27]. Variables were retrieved from archived patients’ registries and analyzed, including baseline demographic data, comorbidities, cardiac arrest etiology, TTM method, complications, and outcomes. TTM-related complications were defined as complications that occurred within 7 days of undergoing TTM. LOS for patients with OHCA was calculated as the time from admission to hospital discharge. LOS for patients with in-hospital cardiac arrest (IHCA) was calculated as the time from the occurrence of the cardiac arrest event to discharge. We defined prolonged LOS as hospital stays beyond the 75th quartile of the entire cohort [28, 29].

A CPC score of 1 to 2 (conscious and alert with adequate or moderate cerebral performance) at discharge was considered a favorable neurological outcome; an unfavorable outcome was defined as a CPC score of 3 to 5 (severe neurological disability, persistent vegetative state, or death) [23, 26, 27].

### 2.3 Statistical Analysis

Categorical variables are described as numbers and percentages. As appropriate, continuous variables are presented as means ± standard deviations or medians with 25th and 75th percentiles (interquartile range [IQR]). Nonrandom associations between two categorical variables were examined using the chi-square test. Continuous variables were tested using Fisher’s exact test for median values or Student’s *t*-test for mean values. One-way analysis of variance with Tukey–Kramer post hoc analysis was used when the means of ≥3 independent variables from the prolonged LOS and non-prolonged LOS groups were compared. All statistical tests were two-tailed, with a *p*-value of <0.05 considered statistically significant.

To predict prolonged LOS, we developed logistic regression (LR)-based models by using patient information at the time of TTM application, post-TTM, and at discharge. Variables were included in LR analysis, and the effects of relevant variables are reported as odds ratios and 95% confidence intervals.

**Fig. 1.**
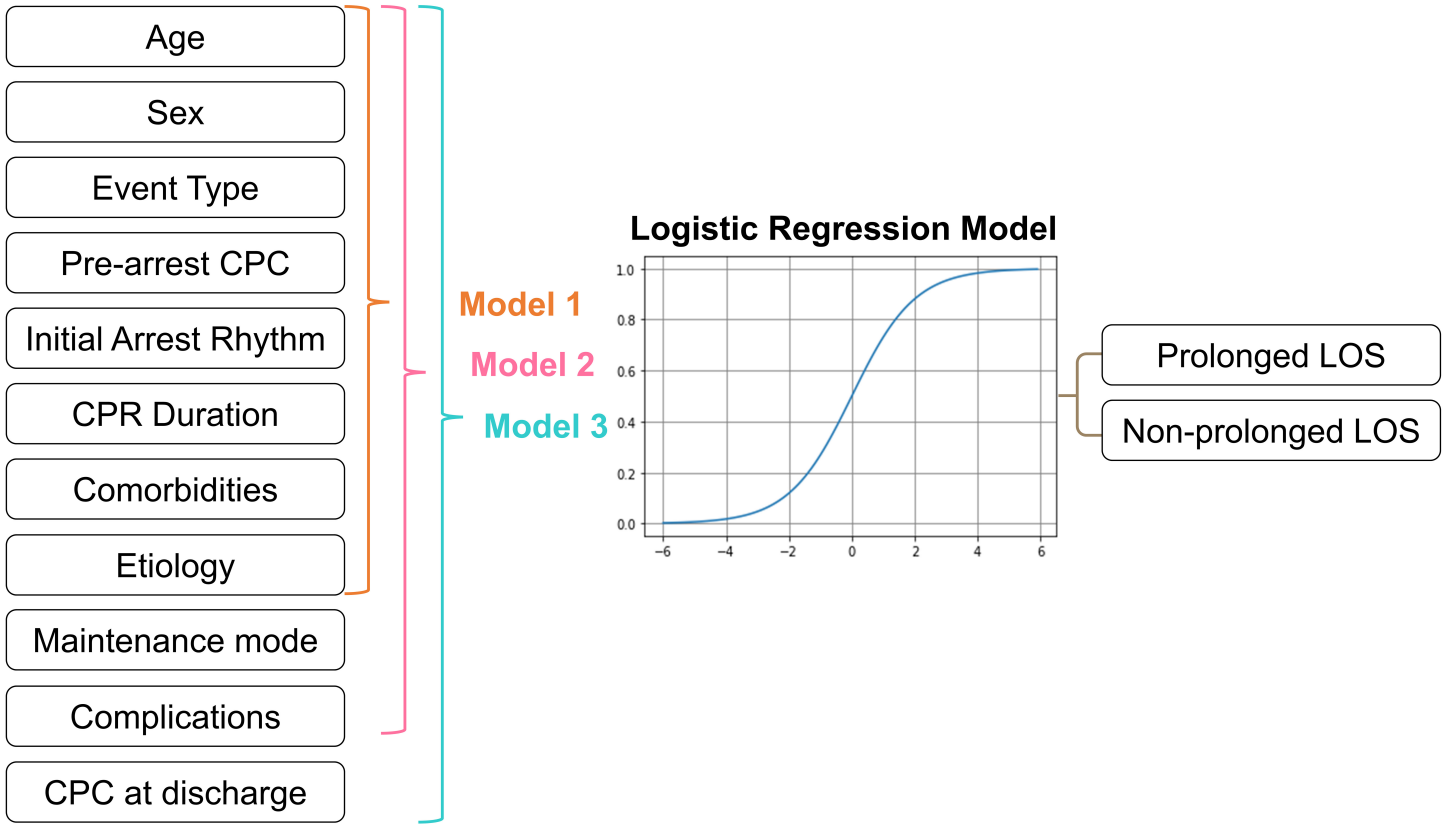
**Logistic regression models predicting prolonged LOS for patients who underwent TTM**. Graphical representation of the predictors and outcomes used in the LR models for predicting prolonged LOS before the initiation of TTM (model 1), after TTM (model 2), and at discharge (model 3). CPC, Cerebral Performance Category; CPR, cardiopulmonary resuscitation; LOS, length of stay; LR, logistic regression; TTM, targeted temperature management.

In model 1, we used patients’ age, sex, event type, prearrest CPC, initial cardiac arrest rhythm, CPR duration, comorbidities, and etiology as predictors, representing the prolonged LOS prediction prior to TTM (Fig. 1). Model 2 contains all the pre-TTM variables in model 1, the maintenance model, and complications of TTM, which represents the prediction after TTM. Model 3 includes all of the aforementioned predictors and the CPC score at discharge, representing the prediction at discharge. The predicted outcome was whether prolonged LOS followed treatment with TTM (Fig. 1). We performed receiver operating characteristic curve analysis and calculated the area under the curve (AUC) to evaluate the level of discrimination of the models. All statistical analyses were performed using STATISTICA v13.3 (TIBCO Software, Tulsa, OK, USA).

## 3. Results

A total of 580 patients were registered in the TIMECARD database during the study period. Nine patients without documented discharge dates from the hospital were excluded from the analysis. A total of 571 patients with cardiac arrest who received TTM (195 women and 376 men; mean age: 64.7 ± 15.9 years) were eligible and were enrolled in this study. Among them, 463 (81.1%) patients had OHCA, and 108 (18.9%) patients had IHCA. The mean and median LOS were 28.8 ± 44.9 and 17 days, respectively, with an IQR of 9 to 35 days for the entire cohort (Table 1). In the entire cohort, 146 (25.6%) patients had prolonged LOS; the mean and median LOS were 73.1 ± 71.1 and 54 days (IQR: 42–69.75), respectively. At hospital discharge, 119 (20.8%) patients had favorable neurological outcomes, and 452 (79.2%) patients had unfavorable neurological outcomes. The mortality rate was 59.2% (n = 338) for the entire cohort. Causes of death after TTM included cardiac event (n = 100, 29.6%), multi-organ failure (n = 66, 19.5%), sepsis (n = 66, 19.5%), respiratory event (n = 59, 17.5%), and others (n = 47, 13.9%, including four patients who were withdrawn from life support after TTM treatment). For patients withdrawn from life support after TTM, the median days from the completion of TTM to the withdrawal of life support was 12 days (IQR: 9–24). For the patients with the shortest quartile of the LOS (less or equal to 9 days), all patients (n = 99) died at hospital discharge.

**Table 1.** **Baseline demographic characteristics of patients according to prolonged LOS in hospital**.

Variable		Entire cohort (n = 571)	Length of stay (LOS) in the hospital		*p*-value
			Prolonged (n = 146)	Non-prolonged (n = 425)	
LOS (days)		17 (9–35)	54 (42–69.75)	13 (6–20)	
Age (years)		64.7 ± 15.9	64.8 ± 15.5	64.6 ± 16.1	0.881
Female, n (%)		195 (34.2)	48 (32.9)	147 (34.6)	0.762
Event type, n (%)					0.0001*
	OHCA	463 (81.1)	102 (69.9)	361 (84.9)	
	IHCA	108 (18.9)	44 (30.1)	64 (15.1)	
Prearrest CPC score		1.29 ± 0.60	1.37 ± 0.68	1.27 ± 0.57	0.087
Initial cardiac arrest rhythm, n (%)					0.886
	VF/Pulseless VT	209 (36.6)	54 (37.0)	155 (36.5)	
	Pulseless electrical activity	138 (24.2)	37 (25.3)	101 (23.8)	
	Asystole	224 (39.2)	55 (37.7)	169 (39.8)	
CPR duration (min)*		24.0 ± 17.7	21.3 ± 14.9	24.9 ± 18.4	0.020*
Etiology					0.201
	Cardiac	301 (52.7)	74 (50.7)	227 (53.4)	
	Asphyxia	116 (20.3)	27 (18.5)	89 (20.9)	
	Sepsis	59 (10.3)	16 (11.0)	43 (10.1)	
	Electrolyte imbalance or acidosis	21 (3.7)	9 (6.2)	12 (2.8)	
	Other medical causes	25 (4.4)	10 (6.8)	15 (3.5)	
	Other nonmedical causes	49 (8.6)	10 (6.8)	39 (9.2)	
Comorbidities, n (%)					
	Hypertension	323 (56.6)	87 (59.6)	236 (55.5)	0.439
	Diabetes mellitus	236 (41.3)	63 (43.2)	173 (40.7)	0.627
	Coronary artery disease	152 (26.6)	45 (30.8)	107 (25.2)	0.194
	Heart failure	109 (19.1)	39 (26.7)	70 (16.5)	0.010*
	Arrhythmia	71 (12.4)	20 (13.7)	51 (12.0)	0.565
	COPD or asthma	62 (10.9)	25 (17.1)	37 (8.7)	0.008*
	Chronic kidney disease	105 (18.4)	36 (24.7)	69 (16.2)	0.026*
	ESRD with dialysis	70 (12.3)	16 (11.0)	54 (12.7)	0.662
	Hepatic insufficiency	18 (3.2)	5 (3.4)	13 (3.1)	0.788
	Previous cerebral vascular disease	75 (13.1)	21 (14.4)	54 (12.7)	0.670
	Hyperlipidemia	106 (18.6)	24 (16.4)	82 (19.3)	0.537
	Malignancy	72 (12.6)	22 (15.1)	50 (11.8)	0.313
Maintenance mode, n (%)					0.774
	Arctic Sun cold blanket	271 (47.5)	73 (50.0)	198 (46.6)	
	Traditional cold blanket	242 (42.4)	57 (39.0)	185 (43.5)	
	ECMO	41 (7.2)	12 (8.2)	29 (6.8)	
	Icy catheter	17 (3)	4 (2.7)	13 (3.1)	
Complications, n (%)					
	Bleeding	156 (27.3)	37 (25.3)	119 (28.0)	0.591
	Arrhythmia	237 (41.5)	54 (37.0)	183 (43.1)	0.207
	Serious infection	257 (45.0)	65 (44.5)	192 (45.2)	0.923
	Seizure	158 (27.7)	55 (37.7)	103 (24.2)	0.003*
	Hypokalemia	363 (63.6)	86 (58.9)	277 (65.2)	0.195
	Hypoglycemia	61 (10.7)	14 (9.6)	47 (11.1)	0.756
Outcome measures					
	CPC score at discharge	5 (3–5)	4 (3–5)	5 (4–5)	0.0004*
	Survival to discharge, n (%)	233 (40.8)	104 (71.2)	129 (30.4)	<0.0001*
	Favorable neurological outcome, n (%)	119 (20.9)	30 (20.5)	89 (20.9)	1.000

COPD, chronic obstructive pulmonary disease; CPC, Cerebral Performance Category; ECMO, extracorporeal membrane oxygenation; ESRD, End-stage renal disease; IHCA, in-hospital cardiac arrest; LOS, length of stay; OHCA, out-of-hospital cardiac arrest; OR, odds ratio; VF, ventricular fibrillation; VT, ventricular tachycardia. *p-value < 0.05. The *p*-value represents the comparison between the prolonged and non-prolonged LOS groups.

### 3.1 Factors Related to Prolonged LOS

In the cohort, a significantly higher proportion of the patients with IHCA had prolonged LOS than did those with OHCA (40.7% IHCA patients with prolonged LOS vs. 22% OHCA patients, *p *= 0.0001; Table 1). The median LOS for the patients with IHCA and OHCA were 25 (IQR: 11–52.75) and 16 (IQR: 9–31) days, respectively (*p* = 0.01). The patients with prolonged LOS had a shorter CPR duration and higher prevalence rates of heart failure, chronic obstructive pulmonary disease (COPD) or asthma, and chronic kidney disease compared with those without prolonged LOS. The patients with COPD or asthma had a longer mean LOS (39.4 ± 5.7 days) compared with those without COPD or asthma (27.5 ± 2 days, *p *= 0.048). The patients who presented with new seizures after TTM were more likely to have prolonged LOS (Table 1).

The factors in Table 1 were included in multivariate analyses to compare the prolonged LOS and non-prolonged LOS groups according to the three models in Fig. 1; Table 2 summarizes the variables with a *p*-value of <0.05 in each of the three models. The multivariate analysis revealed that at the time of TTM, the event type and underlying COPD or asthma were significantly associated with prolonged LOS (model 1). After TTM, the event type, hyperlipidemia, underlying COPD or asthma, and seizures were associated with prolonged LOS (model 2). At discharge, the event type, heart failure, seizure, and CPC score were critical predictors of prolonged LOS (model 3).

**Table 2.** **Multivariable analysis of factors associated with prolonged LOS**.

Variable	Crude odds ratio (95% CI)	Adjusted odds ratio (95% CI)
Model 1		
Event type, IHCA	2.43 (1.56, 3.79)*	2.16 (1.29, 3.64)*
COPD or asthma	2.17 (1.25, 3.74)*	2.35 (1.27, 4.36)*
Model 2		
Event type, IHCA	2.43 (1.56, 3.79)*	2.14 (1.25, 3.66)*
COPD or asthma	2.17 (1.25, 3.74)*	2.46 (1.30, 4.64)*
Hyperlipidemia	1.22 (0.74, 2.00)	1.89 (1.03, 3.50)*
Seizure	1.89 (1.26, 2.82)*	2.25 (1.43, 3.53)*
Model 3		
Event type, IHCA	2.43 (1.56, 3.79)*	2.42 (1.29, 4.54)*
Heart failure	1.85 (1.18, 2.89)*	2.66 (1.30, 4.64)*
Seizure	1.89 (1.26, 2.82)*	1.96 (1.13, 3.41)*
CPC at discharge, 1	1.66 (0.90, 3.09)	2.97 (1.35, 6.55)*
CPC at discharge, 2	4.56 (2.00, 10.40)*	6.48 (2.49, 16.9)*
CPC at discharge, 3	16.66 (7.67, 36.18)*	28.45 (11.62, 69.66)*
CPC at discharge, 4	12.15 (6.94, 21.28)*	16.62 (8.52, 32.43)*
CPC at discharge, 5	1.00	1.00

The table summarizes the variables with a *p*-value of <0.05 in the multivariable analysis of each model. CI, confidence interval; COPD, chronic obstructive pulmonary disease; CPC, Cerebral Performance Category score; IHCA, in-hospital cardiac arrest; LOS, length of stay; LR, logistic regression. *p < 0.05.

### 3.2 Associations between Neurological Outcomes and LOS

**Fig. 2.**
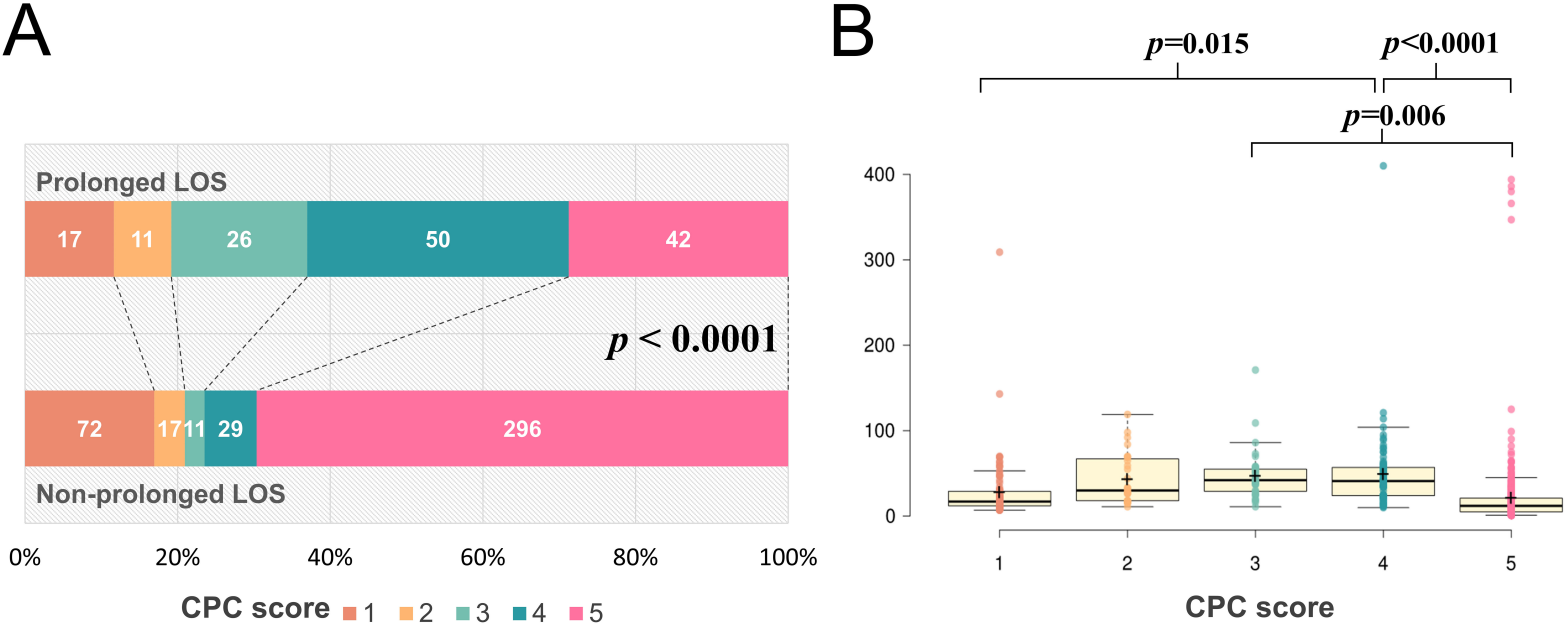
**CPC scores and LOS**. (A) CPC scores of patients with and without prolonged LOS. The difference in CPC scores at discharge for patients with cardiac arrest who underwent TTM with or without prolonged LOS. Each color-coded bar indicates the number of patients. (B) LOS in days for patients with different CPC scores at discharge. The x-axis represents the LOS in days. Box and dot plots indicate the LOS for patients who underwent TTM with different CPC scores at the time of discharge. Data are presented as raw data and box plots with median, 25% quartile, 75% quartile, and standard deviation. Black crosses indicate the mean LOS. Patients with a CPC score of 1 had a significantly shorter LOS than those with a CPC score of 4; patients with a CPC score of 5 had a shorter LOS than those with a CPC score of 3 or 4. CPC, Cerebral Performance Category; LOS, length of stay; TTM, targeted temperature management.

We examined associations between neurological outcomes and LOS in patients with cardiac arrest treated with TTM. A higher proportion of the patients with prolonged LOS tended to survive discharge than did those with a shorter LOS (71.2% vs. 30.4%, *p *< 0.0001, Table 1). Furthermore, the patients with prolonged hospital LOS had a lower CPC score at discharge (Table 1 and Fig. 2A) than did those without prolonged LOS. As illustrated in Fig. 2B, different CPC scores at discharge were associated with LOS (*p <* 0.0001). Post hoc analysis revealed that the patients with a CPC score of 1 had a significantly shorter LOS than did those with a CPC score of 4. The patients with a CPC score of 5 had a considerably shorter LOS than did those with a CPC score of 3 or 4. As depicted in Fig. 2B, the patients with the lowest and highest CPC scores at discharge had a shorter LOS, which may account for the overall favorable neurological outcome and the lack of a correlation with prolonged LOS (Table 1).

### 3.3 Changes in GCS to the Neurological Outcomes and LOS

For the entire cohort, the median time from ROSC to TTM was 4 h 8 min (IQR: 2 h 50 min–6 h 19 min). Most patients (99.6%) showed an improvement in their Glasgow Coma Score (GCS) between the interval following ROSC and initiating TTM, with a GCS of 3.5 ± 1.3 at ROSC and 8.8 ± 1.5 before TTM for the entire cohort. Changes in GCS were associated with neurological outcomes at discharge (*p* = 0.038) but not with prolonged LOS (*p* = 0.424).

### 3.4 Performance of LR Models in Predicting Prolonged LOS

**Fig. 3.**
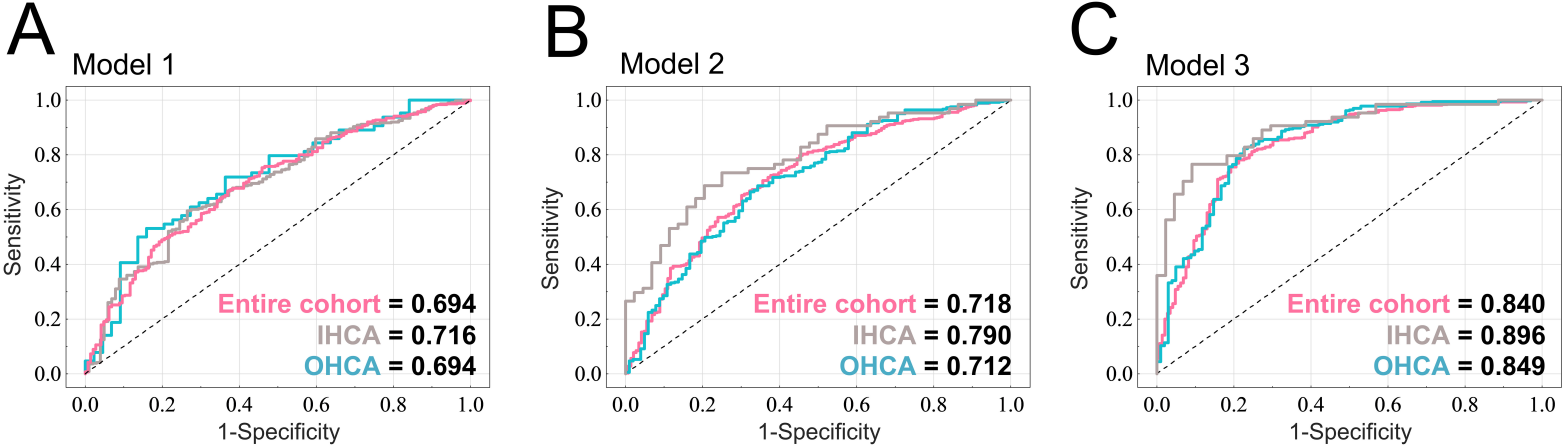
**Performance in predicting prolonged LOS for patients who underwent TTM**. The ROC curves and AUC values of the (A) model 1, (B) model 2, and (C) model 3 for the entire cohort, for patients with IHCA, and patients with OHCA. Models 1, 2, and 3 represent the prediction of prolonged LOS before TTM, after TTM, and at discharge, respectively. AUC, area under the curve; CPC, Cerebral Performance Category; IHCA, in-hospital cardiac arrest; LOS, length of stay; LR, logistic regression; OHCA, out-of-hospital cardiac arrest; ROC, receiver operating characteristic; TTM, targeted temperature management.

As presented in Fig. 1, we developed an LR-based model using patient information before and after the application of TTM, and at discharge to predict prolonged LOS. Fig. 3A illustrates the performance of prediction model 1 for the entire cohort, as indicated by an AUC of 0.694. Furthermore, we also calculated the performance of the LR models for predicting prolonged LOS in patients with IHCA (n = 108) and OHCA (n = 463) who received TTM. The LR model 1 with age, sex, prearrest CPC, initial cardiac arrest rhythm, CPR duration, comorbidities, and etiology as predictors achieved an AUC of 0.716 and 0.694 for the patients with IHCA and OHCA, respectively. Model 2 contained maintenance mode, complications of TTM, and the predictors of model 1, achieving an AUC of 0.718 for the entire cohort. The AUCs of model 2 were 0.790 and 0.712 for the patients with IHCA and OHCA, respectively (Fig. 3B). Model 3 included all the predictors of model 2 and CPC score at discharge, achieving AUCs of 0.840, 0.896, and 0.849 for the entire cohort, patients with IHCA, and patients with OHCA, respectively (Fig. 3C). The acceptable model performance demonstrates the model’s potential to assist in the clinical prediction of prolonged LOS for patients who undergo TTM.

## 4. Discussion

Our findings revealed that, in the current cohort, the median LOS of the patients with cardiac arrest who underwent TTM was 17 days (IQR: 9–35 days). Compared with the patients with OHCA, those with IHCA had a longer LOS. Comorbid heart failure, COPD or asthma, chronic kidney disease, and epilepsy after TTM increase the risk of prolonged hospitalization. The patients who survived cardiac arrest after receiving TTM had longer hospitalization than did those who died, but prolonged LOS was not associated with overall favorable neurological outcomes. At discharge, the patients with a CPC score of 1 or 5 had a significantly shorter LOS, and those with a CPC score of 3 or 4 had a considerably longer LOS. The AUCs of the LR-based prediction model based on patient parameters prior to TTM, after TTM, and at discharge were 0.694, 0718, and 0.840, respectively, indicating that these models exhibited favorable performance in predicting prolonged LOS in the patients with cardiac arrest who received TTM.

TTM was introduced in the 1990s to reduce the metabolic demand on the brain tissue, providing a protective effect for patients after cardiac arrest [30, 31, 32, 33]. However, its effectiveness remains controversial because of the heterogeneity of populations and interventions applied in various studies and the inconsistent results of several trials [34, 35, 36, 37]. Although most discussions have focused on prognoses, our study focused on LOS for patients treated with TTM, which is not linearly correlated with neurological prognosis. The patients with the highest or lowest CPC score had a shorter LOS. By contrast, those who survived to discharge with CPC scores of 3 or 4 had relatively prolonged LOS (Fig. 2B). LOS is an indicator of healthcare resource consumption [5, 38, 39]. A study revealed that the cost of hospitalization for patients who received TTM was 25% more than that for patients who did not receive TTM [5]. Another study comparing historical controls indicated that patients with OHCA who received TTM with unfavorable treatment outcomes (CPC score of 3 or 4) had a longer intensive care unit (ICU) stay and spent more time on ventilators [40]. These results are similar to our findings, and our observations revealed that the patients with a CPC score of 1 or 5 had a shorter LOS. In our study, all the shortest quartiles of hospital stays were those patients who died at discharge. Our results implied that patients with unfavorable prognoses after TTM may require more intensive nursing care and longer hospitalization than did those with favorable outcomes and those who die early after cardiac arrest. On the other hand, sedatives and analgesics are commonly used in patients undergoing TTM. For patients with cardiac arrest, sedatives or neuromuscular blocking agents may be metabolized more slowly [41]. The use and accumulation of these drugs may further cause delayed awakening, thus affecting the prognosis and prolonging LOS in patients who received TTM [16, 18, 41, 42]. In addition to potentially increasing the time spent in the ICU or hospital, TTM may delay critical decision-making regarding whether to continue active treatment, withdraw care, or donate organs [4, 19]. TTM may increase the number of patients who survive cardiac arrest, but these patients may have severely impaired neurological function [34]. Although the patient’s life is prolonged, the subsequent quality of life might be low and potentially costly. Similar to a hypothetical model developed on the basis of the inclusion criteria of the Hypothermia After Cardiac Arrest trial [4], patients who survived but had poor neurological function accounted for the majority of the cost of post-discharge care for patients with cardiac arrest.

In line with the findings of another study [43], our study indicated that the patients with IHCA had a significantly longer LOS after TTM than did patients with OHCA. To our knowledge, no randomized trials of TTM for IHCA have been conducted. A meta-analysis that included six retrospective controlled studies revealed that TTM did not improve survival or neurological function among patients with IHCA [44]. Because the characteristics of patients with IHCA and the treatment received before, during, and after arrest may differ from those of patients with OHCA, the prognosis and LOS after TTM should be evaluated as different entities [37, 43, 45].

In our cohort, receiving TTM and having COPD or asthma as a comorbidity significantly affected LOS. Studies have reported a lower survival rate to hospital discharge and less favorable neurologic outcomes in patients with cardiac arrest comorbid with COPD compared with those without COPD [20, 22, 46, 47]. To our knowledge, no reports on the relationship between COPD or asthma and LOS after TTM have been published. Altered pulmonary compliance resulting from COPD and asthma may lead to respiratory-related comorbidities and complicate treatment applied after TTM, thus affecting neurological outcomes and prolonging LOS [39].

Heart failure is a common comorbidity in patients with sudden cardiac arrest [7, 48]. For patients undergoing TTM, underlying heart failure is associated with reduced survival and a worse prognosis [22, 24]. In our LR model 3, heart failure was demonstrated to be an essential predictor of prolonged LOS at discharge (Table 2). This finding is in line with current knowledge that the characteristics of heart failure include concomitant acute illness and complications, the need for more appropriate treatments, and prolonged hospital stays [1, 49, 50].

In our cohort, 27.7% of the patients developed seizures after TTM and had a significantly longer LOS. The incidence of epilepsy among our patients was similar to that in other reports [51, 52]. Studies have demonstrated an association between seizures and adverse neurologic outcomes in patients after cardiac arrest [41, 51, 52]. The treatment of epilepsy and the use of anesthetic or sedative medications for status epilepticus may result in delayed awakening [53, 54] and prolonged hospitalization of patients with cardiac arrest.

With the continual development of healthcare systems, the management of hospital resources and the ability to predict patients’ LOS are becoming crucial. Our model 3, achieving an AUC of 0.840 to 0.895, provides further relevant knowledge and direction for developing LOS prediction models for patients who receive TTM. With an advanced understanding of patients’ LOS, healthcare teams can make accurate medical decisions, provide information to patients and families regarding expected discharge dates, and design appropriate medical plans [55, 56]. Likewise, LOS is an indicator of the speed of recovery, which can assist patients in the organization and management of their medical budgets. Hospitals can use predicted LOS information to reduce the cost of illness, improve the efficiency of care, and increase the use of resources [55, 56]. Predicting and analyzing factors affecting the LOS of patients with cardiac arrest receiving TTM can improve management and reduce risk factors for patients before and during hospitalization [56].

The correlation between LOS, prognosis, and medical expenditures is multi-factorial and complex. Previous studies have reported that the cost of hospitalization for those patients with cardiac arrest experiencing a CPC of 3–4 was considerably higher than for those with a CPC of 1–2 [39, 57]. Performing early prognostication may help to reduce hospitalization costs. However, when the total long-term costs and benefits for these patients are considered, hospitalization costs are only a small part of the equation. The cost of post-discharge care, rehabilitation, and loss of economic productivity after cardiac arrest can be enormous [2, 3, 4, 5]. Although cardiac arrest patients require considerable cost and resource consumption, this allocation of resources is critical and reasonable when considering the trade-off between inputs and outputs, costs and outcomes in terms of the patient’s long-term survival and quality of life [39, 58, 59].

Our study should be interpreted in the context of the following limitations. First, although this was a multi-center study, the sample size was relatively small. Therefore, a large multi-center cohort with a greater number of patients with different characteristics is required to represent the disease population and validate our results. Second, for patients with IHCA, our registry lacks information about the severity of the disease and events at the time of admission or during hospitalization. The registry also did not have information on whether patients received tracheostomy that required prolonged mechanical ventilation after ROSC. These factors may affect LOS more than the variables reported in the current study. Third, the generalizability of the study may be limited by differences in admission processes and treatment plans among the participating hospitals as well as by the heterogeneity of TTM protocols across the hospitals. Fourth, differences in health insurance, religious affiliation, and socioeconomic status may affect how long a patient remains in the hospital. Our participants were confined to a geographic area and may not be a diverse population. In future studies, different institutions could analyze and develop individualized predictive models based on patient characteristics. Finally, actual medical costs are not reported in the TIMECARD registry. Our study only examined the number of days of hospitalization and could not, therefore, assess the impact of TTM and prognosis on actual medical costs.

## 5. Conclusions

Identification of factors associated with prolonged LOS after TTM, especially at different phases of therapy, can provide essential information that can be beneficial for the medical team while making crucial decisions and designing appropriate medical plans for patients. This information acts as a reference for hospital management for the allocation of necessary treatment resources. Likewise, patients’ relatives can use this knowledge to manage and organize their budgets and expectations of illness.

## Data Availability

The datasets used and analyzed during the current study are available from the corresponding author on reasonable request.

## Abbreviations

COPD, chronic obstructive pulmonary disease; CPC, Cerebral Performance Category; CPR, cardiopulmonary resuscitation; ECMO, extracorporeal membrane oxygenation; ESRD, End-stage renal disease; IHCA, in-hospital cardiac arrest; LOS, length of stay; LR, logistic regression; OHCA, out-of-hospital cardiac arrest; TTM, Targeted Temperature Management.

## References

[1] Benjamin EJ, Muntner P, Alonso A, Bittencourt MS, Callaway CW, Carson AP (2019). Heart Disease and Stroke Statistics-2019 Update: A Report From the American Heart Association. *Circulation*.

[2] Coute RA, Nathanson BH, Kurz MC, DeMasi S, McNally B, Mader TJ (2021). Annual and lifetime economic productivity loss due to adult out-of-hospital cardiac arrest in the United States: A study for the CARES Surveillance Group. *Resuscitation*.

[3] Paratz ED, Smith K, Ball J, van Heusden A, Zentner D, Parsons S (2021). The economic impact of sudden cardiac arrest. *Resuscitation*.

[4] Merchant RM, Becker LB, Abella BS, Asch DA, Groeneveld PW (2009). Cost-effectiveness of therapeutic hypothermia after cardiac arrest. *Circulation: Cardiovascular Quality and Outcomes*.

[5] Damluji AA, Al-Damluji MS, Pomenti S, Zhang TJ, Cohen MG, Mitrani RD (2018). Health Care Costs After Cardiac Arrest in the United States. *Circulation. Arrhythmia and Electrophysiology*.

[6] Girotra S, Chan PS, Bradley SM (2015). Post-resuscitation care following out-of-hospital and in-hospital cardiac arrest. *Heart*.

[7] Chung C, Chiu W, Huang Y, Chan L, Hong C, Chiu H (2021). Identifying prognostic factors and developing accurate outcome predictions for in-hospital cardiac arrest by using artificial neural networks. *Journal of the Neurological Sciences*.

[8] Hypothermia after Cardiac Arrest Study Group (2002). Mild therapeutic hypothermia to improve the neurologic outcome after cardiac arrest. *The New England Journal of Medicine*.

[9] Bernard SA, Gray TW, Buist MD, Jones BM, Silvester W, Gutteridge G (2002). Treatment of comatose survivors of out-of-hospital cardiac arrest with induced hypothermia. *The New England Journal of Medicine*.

[10] Granfeldt A, Holmberg MJ, Nolan JP, Soar J, Andersen LW (2021). Targeted temperature management in adult cardiac arrest: Systematic review and meta-analysis. *Resuscitation*.

[11] Yip Y, Cheung JC (2021). “Cold War”: Why does the debate continue. *Resuscitation*.

[12] Baek H, Cho M, Kim S, Hwang H, Song M, Yoo S (2018). Analysis of length of hospital stay using electronic health records: A statistical and data mining approach. *PLoS ONE*.

[13] Thomas JW, Guire KE, Horvat GG (1997). Is patient length of stay related to quality of care. *Hospital and Health Services Administration*.

[14] Song J, Chen C, Zhao S, Zhou L, Chen H (2021). Trading quality for quantity? Evidence from patient level data in China. *PLoS ONE*.

[15] Hasslacher J, Steinkohl F, Ulmer H, Lehner G, Klein S, Mayerhoefer T (2022). Increased risk of ventilator-associated pneumonia in patients after cardiac arrest treated with mild therapeutic hypothermia. *Acta Anaesthesiologica Scandinavica*.

[16] Taccone FS, Picetti E, Vincent J (2020). High Quality Targeted Temperature Management (TTM) After Cardiac Arrest. *Critical Care*.

[17] Kalra R, Arora G, Patel N, Doshi R, Berra L, Arora P (2018). Targeted Temperature Management After Cardiac Arrest: Systematic Review and Meta-analyses. *Anesthesia and Analgesia*.

[18] Scholte NTB, van Wees C, Rietdijk WJR, van der Graaf M, Jewbali LSD, van der Jagt M (2022). Clinical Outcomes with Targeted Temperature Management (TTM) in Comatose Out-of-Hospital Cardiac Arrest Patients-A Retrospective Cohort Study. *Journal of Clinical Medicine*.

[19] Mulder M, Gibbs HG, Smith SW, Dhaliwal R, Scott NL, Sprenkle MD (2014). Awakening and withdrawal of life-sustaining treatment in cardiac arrest survivors treated with therapeutic hypothermia*. *Critical Care Medicine*.

[20] Chang HC, Tsai M, Kuo L, Hsu H, Huang W, Lai C (2022). Factors affecting outcomes in patients with cardiac arrest who receive target temperature management: The multi-center TIMECARD registry. *Journal of the Formosan Medical Association*.

[21] Golan E, Barrett K, Alali AS, Duggal A, Jichici D, Pinto R (2014). Predicting neurologic outcome after targeted temperature management for cardiac arrest: systematic review and meta-analysis. *Critical Care Medicine*.

[22] Chiu W, Chung C, Huang C, Chien Y, Hsu C, Wu C (2022). Predicting the survivals and favorable neurologic outcomes after targeted temperature management by artificial neural networks. *Journal of the Formosan Medical Association*.

[23] Chou S, Bamodu OA, Chiu W, Hong C, Chan L, Chung C (2022). Artificial neural network-boosted Cardiac Arrest Survival Post-Resuscitation In-hospital (CASPRI) score accurately predicts outcome in cardiac arrest patients treated with targeted temperature management. *Scientific Reports*.

[24] Lin J, Huang C, Chien Y, Hsu C, Chiu W, Wu C (2022). TIMECARD score: An easily operated prediction model of unfavorable neurological outcomes in out-of-hospital cardiac arrest patients with targeted temperaturemanagement. *Journal of the Formosan Medical Association*.

[25] Kołtowski Ł, Średniawa B, Tycińska A, Czajkowska M, Niedziela M, Puchalski W (2021). Predicting survival in out-of-hospital cardiac arrest patients undergoing targeted temperature management: The Polish Hypothermia Registry Risk Score. *Cardiology Journal*.

[26] Chiu W, Lin K, Tsai M, Hsu C, Wang C, Kuo L (2021). Post-cardiac arrest care and targeted temperature management: A consensus of scientific statement from the Taiwan Society of Emergency & Critical Care Medicine, Taiwan Society of Critical Care Medicine and Taiwan Society of Emergency Medicine. *Journal of the Formosan Medical Association*.

[27] Perkins GD, Jacobs IG, Nadkarni VM, Berg RA, Bhanji F, Biarent D (2015). Cardiac arrest and cardiopulmonary resuscitation outcome reports: update of the Utstein Resuscitation Registry Templates for Out-of-Hospital Cardiac Arrest: a statement for healthcare professionals from a task force of the International Liaison Committee on Resuscitation (American Heart Association, European Resuscitation Council, Australian and New Zealand Council on Resuscitation, Heart and Stroke Foundation of Canada, InterAmerican Heart Foundation, Resuscitation Council of Southern Africa, Resuscitation Council of Asia); and the American Heart Association Emergency Cardiovascular Care Committee and the Council on Cardiopulmonary, Critical Care, Perioperative and Resuscitation. *Circulation*.

[28] Chen Y, Scholten A, Chomsky-Higgins K, Nwaogu I, Gosnell JE, Seib C (2018). Risk Factors Associated With Perioperative Complications and Prolonged Length of Stay After Laparoscopic Adrenalectomy. *JAMA Surgery*.

[29] Zhang X, Qiu H, Liu S, Li J, Zhou M (2020). Prediction of Prolonged Length of Stay for Stroke Patients on Admission for Inpatient Rehabilitation Based on the International Classification of Functioning, Disability, and Health (ICF) Generic Set: A Study from 50 Centers in China. *Medical Science Monitor*.

[30] Coimbra C, Wieloch T (1994). Moderate hypothermia mitigates neuronal damage in the rat brain when initiated several hours following transient cerebral ischemia. *Acta Neuropathologica*.

[31] Colbourne F, Sutherland G, Corbett D (1997). Postischemic hypothermia. A critical appraisal with implications for clinical treatment. *Molecular Neurobiology*.

[32] Ginsberg MD, Sternau LL, Globus MY, Dietrich WD, Busto R (1992). Therapeutic modulation of brain temperature: relevance to ischemic brain injury. *Cerebrovascular and Brain Metabolism Reviews*.

[33] Hicks SD, DeFranco DB, Callaway CW (2000). Hypothermia during reperfusion after asphyxial cardiac arrest improves functional recovery and selectively alters stress-induced protein expression. *Journal of Cerebral Blood Flow and Metabolism*.

[34] Dankiewicz J, Cronberg T, Lilja G, Jakobsen JC, Levin H, Ullén S (2021). Hypothermia versus Normothermia after Out-of-Hospital Cardiac Arrest. *The New England Journal of Medicine*.

[35] Lascarrou J, Merdji H, Le Gouge A, Colin G, Grillet G, Girardie P (2019). Targeted Temperature Management for Cardiac Arrest with Nonshockable Rhythm. *The New England Journal of Medicine*.

[36] Nielsen N, Wetterslev J, Friberg H (2014). Targeted temperature management after cardiac arrest. *The New England Journal of Medicine*.

[37] Chan PS, Berg RA, Tang Y, Curtis LH, Spertus JA (2016). Association Between Therapeutic Hypothermia and Survival After In-Hospital Cardiac Arrest. *The Journal of the American Medical Association*.

[38] Stone K, Zwiggelaar R, Jones P, Mac Parthaláin N (2022). A systematic review of the prediction of hospital length of stay: Towards a unified framework. *PLoS Digital Health*.

[39] Petrie J, Easton S, Naik V, Lockie C, Brett SJ, Stümpfle R (2015). Hospital costs of out-of-hospital cardiac arrest patients treated in intensive care; a single centre evaluation using the national tariff-based system. *BMJ Open*.

[40] Storm C, Steffen I, Schefold JC, Krueger A, Oppert M, Jörres A (2008). Mild therapeutic hypothermia shortens intensive care unit stay of survivors after out-of-hospital cardiac arrest compared to historical controls. *Critical Care*.

[41] Panchal AR, Bartos JA, Cabañas JG, Donnino MW, Drennan IR, Hirsch KG (2020). Part 3: Adult Basic and Advanced Life Support: 2020 American Heart Association Guidelines for Cardiopulmonary Resuscitation and Emergency Cardiovascular Care. *Circulation*.

[42] Sandroni C, Nolan JP, Andersen LW, Böttiger BW, Cariou A, Cronberg T (2022). ERC-ESICM guidelines on temperature control after cardiac arrest in adults. *Intensive Care Medicine*.

[43] Chen C, Chen C, Chen T, Yen DH, How C, Hou PC (2020). Comparison of in-hospital and out-of-hospital cardiac arrest patients receiving targeted temperature management: A matched case-control study. *Journal of the Chinese Medical Association*.

[44] Yin L, Xie D, He D, Chen Z, Guan Y, Wang J (2022). Survival to hospital discharge and neurological outcomes with targeted temperature management after in-hospital cardiac arrest: a systematic review and meta-analysis. *Annals of Palliative Medicine*.

[45] Buanes EA, Heltne JK (2014). Comparison of in-hospital and out-of-hospital cardiac arrest outcomes in a Scandinavian community. *Acta Anaesthesiologica Scandinavica*.

[46] Møller SG, Rajan S, Folke F, Hansen CM, Hansen SM, Kragholm K (2016). Temporal trends in survival after out-of-hospital cardiac arrest in patients with and without underlying chronic obstructive pulmonary disease. *Resuscitation*.

[47] Qadeer A, Parikh PB, Ramkishun CA, Tai J, Patel JK (2021). Impact of chronic obstructive pulmonary disease on survival and neurologic outcomes in adults with in-hospital cardiac arrest. *PLoS ONE*.

[48] Hirlekar G, Jonsson M, Karlsson T, Hollenberg J, Albertsson P, Herlitz J (2018). Comorbidity and survival in out-of-hospital cardiac arrest. *Resuscitation*.

[49] Wright SP, Verouhis D, Gamble G, Swedberg K, Sharpe N, Doughty RN (2003). Factors influencing the length of hospital stay of patients with heart failure. *European Journal of Heart Failure*.

[50] Clark KAA, Reinhardt SW, Chouairi F, Miller PE, Kay B, Fuery M (2022). Trends in Heart Failure Hospitalizations in the US from 2008 to 2018. *Journal of Cardiac Failure*.

[51] Mani R, Schmitt SE, Mazer M, Putt ME, Gaieski DF (2012). The frequency and timing of epileptiform activity on continuous electroencephalogram in comatose post-cardiac arrest syndrome patients treated with therapeutic hypothermia. *Resuscitation*.

[52] Knight WA, Hart KW, Adeoye OM, Bonomo JB, Keegan SP, Ficker DM (2013). The incidence of seizures in patients undergoing therapeutic hypothermia after resuscitation from cardiac arrest. *Epilepsy Research*.

[53] Levito MN, McGinnis CB, Groetzinger LM, Durkin JB, Elmer J (2021). Impact of benzodiazepines on time to awakening in post cardiac arrest patients. *Resuscitation*.

[54] Ceric A, May TL, Lybeck A, Cronberg T, Seder DB, Riker RR (2022). Cardiac Arrest Treatment Center Differences in Sedation and Analgesia Dosing During Targeted Temperature Management. *Neurocritical Care*.

[55] Lu M, Sajobi T, Lucyk K, Lorenzetti D, Quan H (2015). Systematic review of risk adjustment models of hospital length of stay (LOS). *Medical Care*.

[56] Abd-Elrazek MA, Eltahawi AA, Abd Elaziz MH, Abd-Elwhab MN (2021). Predicting length of stay in hospitals intensive care unit using general admission features. *Ain Shams Engineering Journal*.

[57] Fukuda T, Yasunaga H, Horiguchi H, Ohe K, Fushimi K, Matsubara T (2013). Health care costs related to out-of-hospital cardiopulmonary arrest in Japan. *Resuscitation*.

[58] Graf J, Mühlhoff C, Doig GS, Reinartz S, Bode K, Dujardin R (2008). Health care costs, long-term survival, and quality of life following intensive care unit admission after cardiac arrest. *Critical Care*.

[59] Sawyer KN, Camp-Rogers TR, Kotini-Shah P, Del Rios M, Gossip MR, Moitra VK (2020). Sudden Cardiac Arrest Survivorship: A Scientific Statement From the American Heart Association. *Circulation*.

